# The Barriers and Facilitators to the Use of Lifestyle Apps: A Systematic Review of Qualitative Studies

**DOI:** 10.3390/ejihpe12020012

**Published:** 2022-01-27

**Authors:** Habiba Shabir, Matthew D’Costa, Zain Mohiaddin, Zaeem Moti, Hamza Rashid, Daria Sadowska, Benyamin Alam, Benita Cox

**Affiliations:** 1 Business School, Imperial College London, London SW7 2AZ, UK; matthew.dcosta20@imperial.ac.uk (M.D.); zain.mohiaddin20@imperial.ac.uk (Z.M.); zaeem-zunaid.moti20@imperial.ac.uk (Z.M.); hamza.rashid17@imperial.ac.uk (H.R.); daria.sadowska20@imperial.ac.uk (D.S.); b.cox@imperial.ac.uk (B.C.); 2 The Queen Elizabeth Hospital Birmingham, Mindelsohn Way, Birmingham B15 2GW, UK; benyamin.alam@uhb.nhs.uk

**Keywords:** mobile health, mHealth, health prevention, health promotion, applications, lifestyle apps, health interventions, smartphones

## Abstract

Background: Mobile-health applications are revolutionising the way healthcare is being delivered. However, current research focusses on apps aimed at monitoring of conditions rather than the prevention of disease. Healthcare apps that prevent disease can be classified as lifestyle apps (LAs) and encompass mindfulness, exercise, and diet apps. In order for widespread implementation of these apps, perspectives of the user must be taken into consideration. Therefore, this systematic literature review identifies the barriers and facilitators to the use of LAs from a user’s perspective. Objective: To both identify the facilitators to the use of LAs from a user perspective as well as identify the barriers to the use of LAs from a user perspective. Methods: A systematic literature review was conducted following PRISMA guidelines. Qualitative articles focussed on a healthy non-diseased population were obtained. Two independent researchers coded the articles, and themes were identified. Results: Our results found that there were five barriers and five facilitators to app use. The facilitators included (1) motivational aspects to the user, (2) effective marketing and communication, (3) user-centred design and content, (4) humanising technology, and (5) accessibility. The five barriers identified were (1) a non-conducive, (2) poor marketing and branding, (3) controlling and invasive, (4) disengaging content, and (5) inaccessibility. Conclusions: By overcoming the barriers of LAs and encouraging the facilitators found, users are more likely to engage with this method of health promotion. Future research must be conducted on the barriers and facilitators to development and distribution of apps in order for LAs to be implemented in widespread healthcare practice.

## 1. Introduction

With 3.8 billion smartphone users worldwide [1], mHealth apps represent the largest and most-significant manifestation of mHealth in the 21st century [2]. mHealth apps can be defined as “softwares that are incorporated into smartphones to improve health outcome, health research, and health care services” [3]. Compared to other health interventions channelled through laptops and computers, mHealth apps represent a cheaper and therefore more-accessible platform.

**Figure 1:**
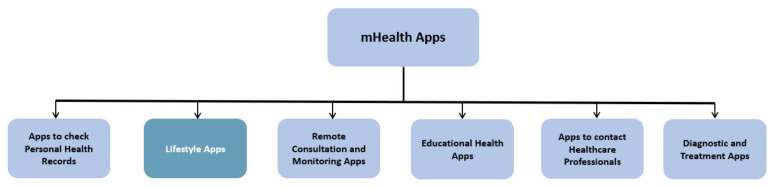
A summary of the various types of mHealth apps. Adapted from [4].

There are various categories of mHealth apps in healthcare practice. These are summarised in Figure 1.

**Figure 2:**
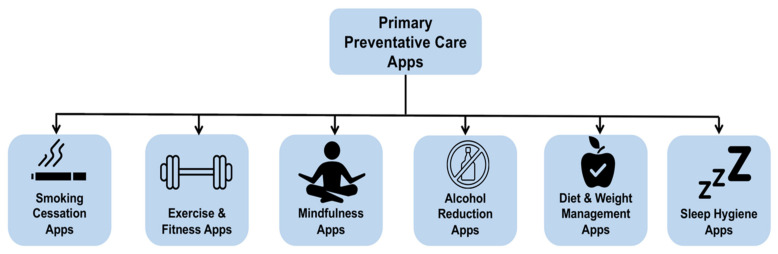
Examples of primary preventative-care apps.

Our SLR focusses on fitness and well-being apps summarised as lifestyle apps (LAs) that aim to prevent users from acquiring disease. Examples of these apps are summarised in Figure 2.

Rationale for conduction of SLR:

Lifestyle and wellbeing apps comprise two-thirds of all mHealth apps on the market [5]. However, studies on these apps, which are dominating the market, are sparse [6]. Lifestyle apps offer an excellent way to encourage prevention-based care rather than the current method, focusing on the treatment of conditions, which is more expensive and which reduces the quality of life of an individual [6]. By improving involvement and participation with these preventative apps, public health could be greatly improved, and fewer would need to resort to seeking healthcare services for treatment. It is clear lifestyle apps are an effective form of health promotion, but there is currently little research on how the use of LAs can be propagated. Therefore, the primary objective of our systematic literature review (SLR) was to explore the barriers and facilitators to the use of lifestyle apps from a user perspective.

In this context, “use” encompasses three aspects:

- Adoption: This concerns the ability of LAs to acquire new users.
- Engagement: This describes the extent to which users interact with lifestyle apps.
- Retention: This factor is strongly interlinked with engagement and describes users returning to lifestyle apps, after initial use.

## 2. Methods

### 2.1. Search Strategy

The topic was formulated into a structured research question via the SPIDER framework [7], shown in Table 1. The Boolean operators were then used to formulate these into a search string (Table A1 in Appendix A).

**Figure 3:**
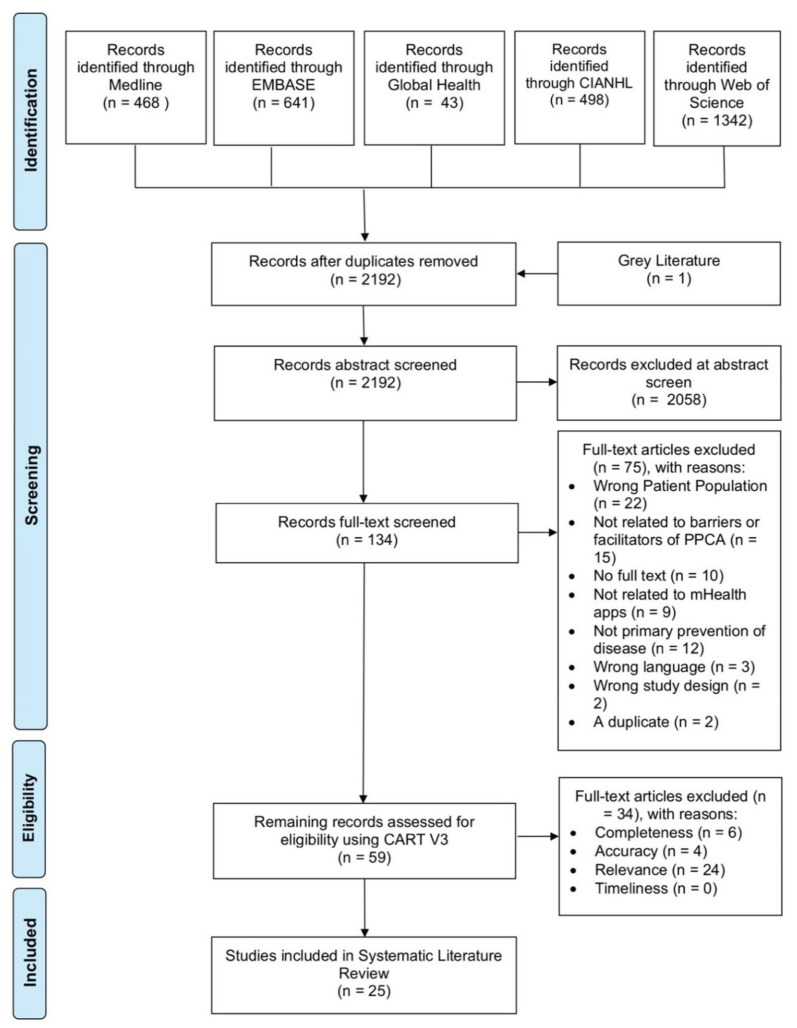
A PRISMA flow diagram illustrating the search results.

Five databases (MEDLINE, EMBASE, Global Health, CINAHL, and Web of Science) were searched whilst adhering to the Preferred Reporting Items for Systematic Reviews and Meta-Analyses statement (PRISMA) guidelines [8]. See Figure 3 for the exact procedure undertaken. Three search limits were set: articles written in English, articles with an abstract available, and articles published after 2016. The latter was chosen due to the surge in mHealth-app developers from 2016 onwards [9]. The aforementioned search string was used to find a total of 2992 articles. Furthermore, grey literature was also searched for to reduce publication bias.

### 2.2. Inclusion and Exclusion Criteria

The inclusion and exclusion criteria were decided amongst the authors of this project to minimise bias; this is illustrated below, in Table 2. Only studies conducted on a healthy population were included, as clinical populations were deemed to have different behavioural and psychological drivers that influenced their app use [10].

### 2.3. Search Results

The 2992 articles found wer then reduced to 2192 following the removal of duplicates. Using the exclusion criteria (Table 2), six independent reviewers performed abstract and title screening. Four independent reviewers then proceeded to perform the full text review. Articles were only included if they met the inclusion criteria described in Table 2. Each article was also assessed based on the CART framework [11] (Table A2 in Appendix A). This process is outlined below using the PRISMA flow chart (Figure 3). The final number of articles included in the review was 25.

### 2.4. Data Synthesis

A coding scheme was designed by two reviewers to extract data from the 25 articles (Table A3 in Appendix B). From these codes, inductive reasoning was used to develop themes concerning the barriers and facilitators of the use of mobile-health apps in the primary prevention of disease.

### 2.5. Critical Appraisal

CASP is a systematic method of evaluating the strengths and limitations of qualitative research, assessing them based on a criterion of trustworthiness, value, and relevance [12]. Two independent reviewers critically appraised each study using CASP.

## 3. Results

### 3.1. Facilitators

**Figure 4:**
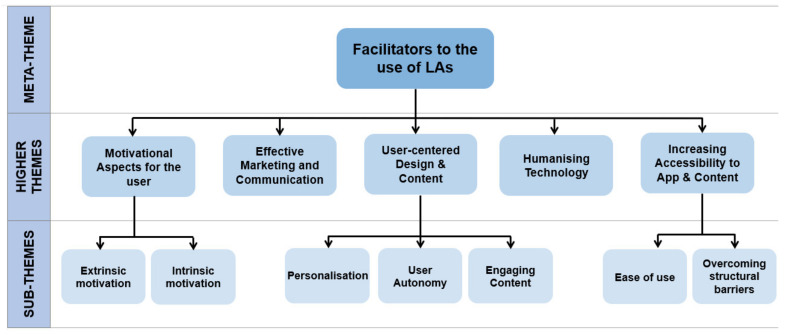
A thematic map of the facilitators to the use of LAs, classifying these variables from broad ideas to specific and individualized concepts.

From the 25 articles reviewed, five higher themes and seven sub-themes were identified as facilitators to the use of LAs. These are outlined in Figure 4 below.

#### 3.1.1. Theme 1: Motivational Aspects for the User

##### Intrinsic Motivation

“*People are motivated from the inside*”

[13]

Intrinsic motivation is essential to ensure the continued use of Las [2,13]. Zhang [13] found that users are “motivated from the inside,” as well as externally. As usage of LAs is not compulsory, some level of intrinsic motivation must be present within the user to ensure continued use [2]. It was also identified that the development of routines was a method by which intrinsic motivation could be fostered as “the momentum” to continue using LAs could be more easily generated [14]. Moreover, intrinsic motivation usually drives greater self-discipline, increasing user retention [15].

##### Extrinsic Motivation

*“Even if it’s just a reminder. It pushes us through”*

[16]

Additionally, the existence of extrinsic motivation within the LA and the environment of the user is also important to facilitate continued use.

Gamification and other reward-based systems have been identified as important features that motivate users, especially younger audiences, to use LAs [15,16,17]. Both the ability to compare oneself against their former self and the rewards that these systems offer are engaging for users. The rewards used in these systems should be tangible and varied, with some studies even suggesting that financial incentives could be motivational for users [15,16,18].

Moreover, the act of goal setting and the subsequent tracking of progress can be an effective method of motivating LA users [16,18,19]. However, LAs should not just track progress but also interpret and analyse the data collected, offering useful and thought-provoking advice to best motivate users [20].

The presence of leader boards that compared users against one another encouraged the use of LAs, because users had the desire to “beat” their friends [16,18,21]

Finally, the existence of supportive and active social environments helps users to see the benefits of LAs more clearly and therefore leads to greater user engagement with the app [22,23]. Furthermore, support members using the LA alongside the participant nurtured user engagement further [14].

#### 3.1.2. Theme 2: Effective Marketing and Communication

“*I did not go into something that I did not know*”

[24]

As with all products, an effective marketing strategy such as the communication of the key benefits, the brand associated, and the presence of the LA within the app store is key to adoption.

The promotion of the LA characteristics, such as the existence of “evidence-based” content from “trustworthy” sources within the app, has been demonstrated as paramount to LA uptake [23,25,26]. This communication is essential for paid LAs, as users need to clearly understand the value for money of such apps to adopt them [15,24]. It was also found that users are significantly influenced by word-of-mouth recommendations from family and friends [15,24,27,28,29].

Brand awareness was crucial to successful adoption as users were more inclined to try apps that came from reputable and well-known organisations. [27,29,30]. Moreover, brand presentation was seen as a key method by which the benefits and functions of the LA could be communicated to the user, encouraging further uptake [6,15,27,28,30].

Finally, placement in the app store was important in determining user uptake. Apps that were found higher up in the search results were deemed by users to be of a higher quality and attracted more attention [29].

#### 3.1.3. Theme 3: Increasing Accessibility to Apps and Content

##### Ease of Use

“*You want the process to be as simple as possible*”

[15]

Zhang and Xu (2020) demonstrated that ease of use was one of the “most crucial factors” that determined user retention (*p* < 0.001). This ease of use can be fostered through the development of intuitive and simple interfaces that allow users to access the content in a “smooth[er]” way [15,21,28]. The incorporation of visual aids, such as pictures, can make the contents of the LA easier for users to understand [23]. Features that conveyed information in a more concise and understandable manner reduced the time and effort needed to use LAs [10,19,27,30].

##### Overcoming Structural Barriers

“*I just looked at the word straight away and I was like I can’t read that, I’m not going to even try*”

[30]

The Urban Institute (2015) defines structural barriers as obstacles that collectively affect a group disproportionately and perpetuate or maintain stark disparities in outcomes. The SLR commonly identified multiple ways in which these barriers could be overcome, increasing user accessibility to LAs:

Reigner et al. (2018) found that often users from lower socioeconomic groups have less disposable income to spend on smartphones and on WIFI, meaning that their access to LAs is limited significantly [23].

Users expressed the need for larger icons and the ability to adjust font size as key to helping elderly users engage with the app [27,31]. Similarly, Castro et al. and Brewer et al. emphasized the need for the language used within the app to be easy to comprehend so that lower-educated groups, who are typically at a greater risk of lifestyle-related disease and therefore have the most to gain from such interventions, can still access LAs [16,27].

Many do not find the advice given in LAs as relevant to their daily lives due to their lack of cultural inclusivity. The LA used in the Brewer et al. (2019) study had an increased appeal to diverse minority communities as they included “ethnic cuisines,” which were more relevant to the cultures of these groups. Similarly, Brewer (2019) also found that these users responded better to images that were more representative of them [27].

#### 3.1.4. Theme 4: User-Centred Design and Content

##### Personalisation

“*Not everyone is auditory, not everyone is visual, not everyone is kinaesthetic…*”

[14]

Laurie and Blandford (2016) found that users are of different abilities and therefore want information and guidance that is personalised to their specific situation, skillset, and preferences [14]. Furthermore, users often have multiple needs that they are trying to address; therefore, multifunctional apps tend to be more popular than apps with singular functions [10,28,32]. Moreover, users respond differently to different communication methods within LAs (e.g., visual, auditory, and kinaesthetic), and therefore, the communication must also be customised to the user’s preferences [10].

Information should also be tailored and made relevant to individual users [15]. This can be supported to a large extent by ensuring that during the adoption phase, users choose apps that align with their preferences, personality, and expectations [2]. If there is alignment between the user’s expectations of the LA and the app’s actual functions, then users’ interactions with the app are likely to be of a higher quality [14].

As the majority of LAs tend to implement behaviour changes, a substantial amount of research supports the delivering of structured action plans, tailored to the user’s personal challenges and goals [2,10,20]. The setting of specific goals, the monitoring of progress, and the receiving of relevant feedback based on user data all serve to make LAs more personalised [2,3,10,24,26,28,30,31]. These features allow users to adjust their goals and activity accordingly, thus promoting engagement. Personalised notifications and reminders increase the probability of users adhering to such plans [17,26,28,30,33]. High-quality, relevant, and non-generic feedback stimulates users to reflect upon their actions, hence increasing subsequent engagement [2,10,13].

##### User Autonomy

“*I don’t want to do X km because the app says so, but because I want to*”

[10]

Users want autonomy over how and when the app should be used. Peng et al. (2016) noted a profound dislike concerning the concept of “taking orders from a machine” [15]. If app usage is self-directed, users are more likely to frame their interactions with the app as something they “want” to partake in, rather than an activity that they “have” to partake in, encouraging further use of the app [14,31]. Users also desire ultimate control over their data, especially when choosing which parties can have access to it [15,21,24].

The ability to customise features to the user’s preferences can also support user autonomy [18,19]. For instance, as users respond differently to push notifications, they should be able to control the frequency and the type of notifications they receive, to better support their interactions with the app [2,13,14,26].

##### Engaging Content

“*It’s got to be different everyday*”

[20]

User retention is a difficulty that several LAs face and therefore, to remain engaging over long periods of time, it is important that the content of the app is dynamic and frequently updated [29].

Furthermore, if information is more practical, then user-behaviour change is more likely to occur as they receive more actionable advice [17]. This increases the likelihood of users seeing benefit in the application and their subsequent uptake of the LA. The accuracy of content is essential to ensure the continued use of such apps as it determines user trust and subsequent long-term adoption. [2,6,13]. For this reason, the existence of evidence-based content within LAs is essential [17,19,27]. Moreover, through the provision of animation, videos, and gamification, developers can deliver content in a more engaging way, increasing user engagement and retention.

#### 3.1.5. Theme 5: Humanising Technology

“*For me, it should be…a bit more human*”

[10]

LAs require a level of genuine human interaction to effectively engage users. Human interaction within LAs was important for many users as when apps felt impersonal, users often felt “feelings of loneliness,” reducing their engagement [21]. Participants of several studies desired the app to feel like a “friend,” with messages being delivered by “real people” as this was more motivational than artificial interfaces [14,16,26,27]. Similarly, involving “success stories,” ex-users who had successfully responded to the intervention in the LA, was found to inspire and motivates users, as often they found the advice given by these ex-users to be empathetic and relatable [20].

### 3.2. Barriers

**Figure 5:**
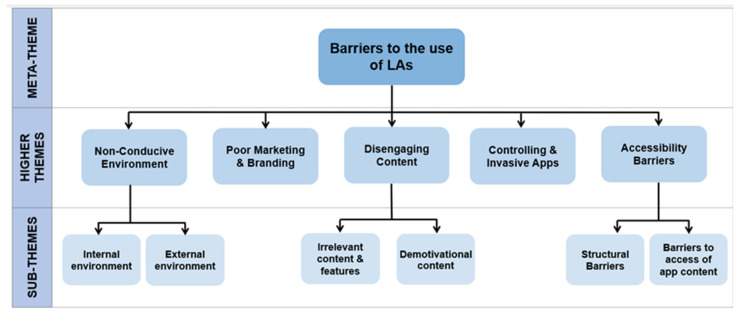
A thematic map of the barriers to the use of LAs, stratifying these obstacles from over-arching concepts to specific and individualized hurdles.

From the SLR, a total of five higher themes and six sub-themes were identified with relation to the barriers to the use of LAs. These are summarised in Figure 5.

#### 3.2.1. Theme 1: Accessibility Barriers

##### Structural Barriers

“*I don’t think my telephone is good enough… I will change it, but I don’t know when.*”

[23]

Interestingly, the cost of the app is a barrier that prevents the adoption of LAs across a diverse range of demographics spanning “all participants in all age groups and socioeconomic status” [15]. However, it disproportionately affects poorer socioeconomic groups and reduces their usage the most. These users are more likely to have low-end phones that cannot support the LA’s functions [16].

Digital illiteracy is another barrier to the use of LAs [21]. Users who are digitally illiterate often do not have the knowledge, skills, or ability to use LAs, meaning that the benefits they can gain from such interventions are limited [23].

In addition, the lack of availability of LAs in multiple languages directly discriminates against non-native speakers [23]. Similarly, the use of complex and confusing terminology within LAs can make them inaccessible to lower-income groups or those with dyslexia and reading difficulties [28,30]. The layout of content can act as a barrier to the use of LAs as users with dyslexia and reading difficulties often find dense text confusing and prefer information in a more-concise format with less jargon [17,19,27,30].

##### Barriers to Access of App Content

“*A lot of functions are everywhere. I don’t know where to go, how I can change the functions, and where to go…*” [18]

Users disliked over-complicated apps with irrelevant features and clustered interfaces, often feeling “overwhelmed” by them. [15,16,17,18,21,28]. Even menial tasks such as logging in to the LA caused disengagement when such tasks were too lengthy [23].

#### 3.2.2. Theme 2: Poor Marketing and Branding:

“*People just don’t know what is*”

[17]

LAs that did not have strong enough advertising campaigns were unlikely to be adopted. If marketers did not promote the app enough, it was unlikely to attract user attention. Peng et al. 2016 noted that up to 25% of consumers were not even aware that mobile-health apps existed, demonstrating that a lack of targeted promotion was prevalent throughout the industry. Consumers often weigh the potential benefits of the LA against risks such as the cost of the app and the app’s battery and storage consumption. Therefore, firms must clearly promote the benefits of their LAs and design their products in ways that reduce the risk for users (e.g., by reducing the size of the app).

#### 3.2.3. Theme 3: Controlling and Invasive Apps

“*I don’t want an electronic device telling me what to do*”

[6]

The presence of patronising and authoritarian language within LAs was deemed demotivating and thus a barrier to their use [19,33]. Authoritarian connotations were further illustrated by “pre-set” app features and were disliked as it encroached upon the users’ freedom to choose how and when they used the app [10].

Users were also deterred by notifications and advertisements that were deemed too invasive [10,21,25,26,28,32,34]. For this reason, Peng et al. (2016) and Nour et al. (2019) recommended the timing and frequency of notifications to be well-designed and customizable to the users’ schedule. Similarly, unnecessary, invasive questions such as requesting “irrelevant” personal information or access to apps and users’ contacts was considered a significant barrier to the use of LAs [15,21].

Users were reluctant to share data due to “fear of exposure”; this was especially true for users who did not have sole possession of their devices but instead shared them with other close social members of their social network [10,23]. However, others relayed that if the perceived benefits were greater than the risk of data leakage, they would use the app [34].

#### 3.2.4. Theme 4: Disengaging Content

##### Irrelevant Content and Features

“*What is your point…. you don’t need to tell me*”

[30]

LAs that had irrelevant and insufficient content were seen as being of poorer quality and less credible, resulting in lower user engagement [2,14,17]. Moreover, many authors including Lieffers (2018) found that users experienced frustration when an app did not give them the ability to customize the app’s features to meet their preferences.

##### Demotivational Content

“*It makes me feel somehow bad*”

[32]

Additionally, users often felt demotivated by the negative framing of concepts and feedback used in LAs, leading to disengagement. Despite occasionally being motivational for individuals who lacked self-discipline [14], most of the time, users responded to negative feedback by withdrawing from the app [15,28,32]. Similarly, when scientific evidence for behaviour change was framed in a negative light, users felt threatened and were demotivated to use the LA [17].

#### 3.2.5. Theme 5: Non-Conducive Environment

##### External Environment

“*When I feel that I have to do too many things, I feel that I cannot fit the space to do it*”

[14]

Rose (2020), Reginer et al. (2018), and Laurie and Blandford (2016) reported that users often have busy and demanding lifestyles and therefore less time available for using LAs. Furthermore, busy schedules often lead to the establishment of “psychological effects” whereby the user feels greater stress and perceives the app as a chore, decreasing the likelihood of continued use [14,23,26]. Additionally, busy lifestyles often restricted the capacity of the user to gain from some of the LA’s benefits [16,18,19].

##### Internal Environment

“*If you’re feeling quite stressed, probably using the app is going to be harder*”

[14]

The user mindset was identified as key to determining whether users remained engaged with their LA and was acknowledged to be particularly influenced by the user’s personal life and emotions. Stressed or agitated users were less likely to successfully use LAs [14]. Additionally, several authors explored how users often withdrew from LAs because they felt the apps had begun to take over their lives [23,28,32]. For instance, Rose (2020) described the adverse effects of user addiction after using LAs too much. Users also admitted to being distracted by other apps on their devices, leading to reduced engagement with the LA.

## 4. Discussion

The new horizons mHealth technologies offer are changing the landscape through which healthcare is delivered. To the best of our knowledge, this study is one of the first that looks into barriers and facilitators to the use of primary preventative-care apps from a user’s perspective. Most of the current research on mHealth adoption is from patients’ perspectives. In contrast, the target group for LAs is not exclusive to patients but rather encompasses the whole population [6].

The list of barriers and facilitators identified were categorised into 10 main themes.

The facilitators included: (1) *motivational aspects to the user* (i.e., the user must have intrinsic and extrinsic sources of motivation to use the app), (2) *effective marketing and communication* (i.e., the user must be aware of the app and its benefits) (3) *user-centred design and content* (i.e., the app must be personalised to the users’ needs), (4) *humanising technology* (i.e., the app must be empathetic and compassionate), and (5) *accessibility* (i.e., the app must be easy to use and available to all).

The five barriers identified were: (1) *a non-conducive environment* (i.e., the users’ environment prevents access of apps), (2) *poor marketing and branding* (i.e., the communication of the app is not relayed to the user), (3) *controlling and invasive apps* (i.e., the app invades users privacy and is regimented), (4) *disengaging content*, and (5) *inaccessibility* (i.e., an inability to either access the app or use it).

The primary function of LAs is to prevent users from acquiring disease. One of the main barriers identified to their use was that of a lack of accessibility. This barrier is especially pertinent as it disproportionately hinders lower socioeconomic demographics from using them. These are individuals who are traditionally in the greatest need and stand the most to benefit from such preventative measures—it is well documented that low socioeconomic groups smoke more, exercise less, and are twice more likely to be obese compared to higher socioeconomic groups [35,36]. Consequently, they are more likely to develop chronic diseases such as cardiovascular remodelling and type-2 diabetes in later life. LAs have been proposed by several parties as a “cheap and widely available tool” to deliver preventative care to more deprived areas [37]. Therefore, engaging app developers about the importance of developing affordable LAs or for healthcare systems to adopt subsidisation models that reimburse users paying for verified, high-quality applications and smartphones can perhaps aid the adoption of LAs as a preventative measure for such communities. However, with an increasing deficit faced by the NHS, rigorous cost-effectiveness studies to weigh the benefits of subsidation of LAs and smartphone-related costs by the NHS against their significant expense must be performed. This is one dimension of value-based healthcare, which the NHS defines to be the “equitable, sustainable and transparent use of the available resources to achieve better outcomes and experiences for every person” [38]

Furthermore, the wide variety of opinions on the barriers and facilitators to LA use was consistent throughout our research. For example, opinions varied regarding the optimal number of features in apps. Some preferred a large multi-functional app that had a broad selection of features available, preventing the need to download multiple apps. However, others preferred an app with few features as they reported this as being easier to use, which is in accordance with the reduction principle laid out by Kukkonen and Harjumaa’s Persuasive System Design Framework (2008). These serve as strong indicators for the need to tailor LAs to individual preferences. This could be achieved by developing a questionnaire for users, allowing identification of these preferences and subsequent personalisation of LAs through the use of this information, as suggested by Simons et al. [19]. 

In this review, motivation was identified as a key facilitator to the use of LAs. As outlined in the Section 3, motivational aspects identified fell into two categories—external and internal. Whilst an in-depth analysis of the relative motivational aspects contributing to prolonged LA use was not conducted, many studies highlight the importance of both elements being present for the effective and continued use of LAs. As LA use is completely voluntary, the presence of self-motivation is fundamental and arguably more important than external features. Previous research has shown that when there is no mandate to enforce the use of a technology, people have a lower tendency or positive attitude toward using that technology [39], unless internally motivated to do so [40]. In such a situation—common to mHealth apps—those who have higher persistence toward reaching their goals (stronger intent) appear to have longer continued engagement with the technology [2].

### Limitations of Systematic Literature Review

The findings of the SLR were subject to several limitations that restricted their generalisability. Firstly, despite being written amidst the COVID-19 pandemic, the decision was made to exclude articles that focused solely on the effects of the pandemic on the uptake of LAs. This decision was taken due to the uncertainty that continues to surround the pandemic and its impact on mHealth-app usage. However, it should be recognised that the pandemic has had a significant impact on consumer behaviour concerning digital health, especially for fitness and meditation apps [41,42,43]. Therefore, failing to acknowledge that the pandemic will impact future studies concerning the facilitators of mHealth apps would be a form of gross negligence on the part of the reviewers [34].

Another limitation that reduces the generalisability of the data analysed in the SLR was that only articles written in English were included. Therefore, there was a disproportionate number of studies, within the review, that were conducted in MEDCs, meaning that these findings are unlikely to be transferable to LEDCs, where structural barriers such as illiteracy and inaccessibility to smartphones may be more of a barrier to the use of LAs. Furthermore, due to the limited number of UK-based studies concerning the barriers and facilitators of LAs, the SLR had to include studies from elsewhere around the world, meaning that the findings were not entirely generalisable to a UK population either.

Finally, it should be recognised that as all of the included studies were conducted on a healthy population, the results are not generalisable to populations with illness. The existence of prior disease or comorbidities is likely to affect a user’s use of a preventative-care app, and this needs to be acknowledged when considering how to implement mHealth apps in the context of healthcare [10].

This review focussed on apps focussed on the prevention of disease, which have significantly risen in use during the COVID-19 pandemic [39]. However, much of the current literature still revolves around apps focussed on the management of diseases.

Although this review identified the users’ perspectives on the adoption of LAs, for the wider implementation of LAs in healthcare, “use” cannot be looked upon in isolation. There are also many external upstream factors influencing it, for example, the development and delivery of apps. Future research should therefore address the barriers and facilitators to the development of apps (i.e., the processes involved in the creation of LAs by app developers, software engineers, and designers) and the distribution of LAs (i.e., the processes involved in the provision of LAs to the end-user, of which is heavily influenced by HCP’s) in order for holistic implementation to be achieved.

## 5. Conclusions

Mobile-health apps are one of the most-significant manifestations of medical technology in the 21st century. LAs in particular can be considered as the most-accessible behaviour-change intervention to the general public and therefore a helpful tool to promote health and prevent disease. Hence, to encourage the implementation of these valuable apps, an SLR was conducted, which identified five barriers and five facilitators to the use of LAs from a user perspective. Whilst this literature review offers a comprehensive understanding of the barriers and facilitators to the use of LAs, due to 19 out of 25 of the studies being conducted abroad, the application of our findings may not necessarily apply to the UK. Furthermore, as the articles included focussed exclusively on a healthy population, in order to target the general population, the barriers and facilitators for app use in a diseased population should also be investigated. It is likely these will vary from those found in this study due to different motivational factors and psychological effects of disease. Moreover, future research should also aim to understand the barriers and facilitators to the development and distribution of LAs to the end user to understand the full life-cycle when incorporating LAs into healthcare practice.

### Implications

With the significant advancement and availability of technology, lifestyle apps pose an excellent way to transform the scope of preventative healthcare. Therefore, in order to support this increasing trend of lifestyle-app use, this study identified the greatest obstacles, which need to be addressed in order for the success of mobile-health technology. These aforementioned hurdles can be overcome through several methods as proposed in the discussion. Furthermore, this study identified methods to facilitate lifestyle-app use. By identifying both the facilitators and barriers to lifestyle-app use from a user perspective, these can be promoted and addressed, respectively, increasing user adoption of lifestyle apps and hence reducing the disease burden on the healthcare system.

## Figures and Tables

**Table 1:** The SPIDER framework used for the SLR.

Sample	Smartphone users
Phenomenon of Interest	LA use
Design	Surveys and Interviews
Evaluation	Barriers and Facilitators
Research Type	Qualitative

**Table 2:** An outline of the SLR inclusion and exclusion criteria.

Inclusion	Exclusion
Focuses on LAs	Doesn’t focus on LAs
Primary Research	Review articles or any form of secondary research
Published between 2016 and 2020	Published before 31 December 2015 or after 1 January 2021
Full article published in English	Article not published in English
Focuses on the barriers or facilitators to the use of LAs	Doesn’t focus on the barriers or facilitators to the use of Las
Qualitative Research	Quantitative Research
	Focus on the use of short message services or mHealth apps solely used for surveillance or location tracking (e.g. Reminders for patient checkups)

**Figure A1:**
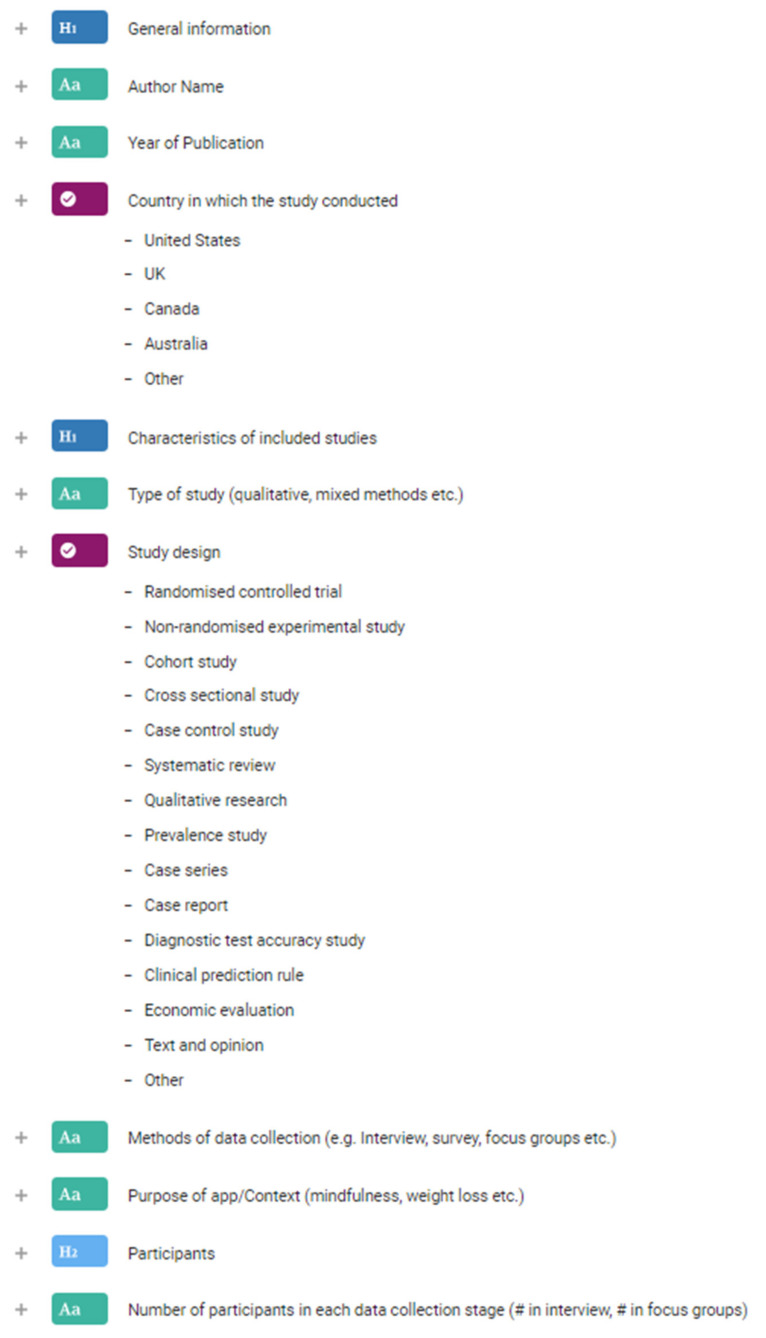
Data Extraction Template.

## Data Availability

Not applicable.

## Appendix A. Systematic Literature Review

**Table A1:** Search String.

SPIDER	Search String
Sample	digital apps or digital applications or digital app or digital application or medical apps or medical applications or medical app or medical application or mHealth or mHealth app or mHealth application or mHealth apps or mobile health or smartphone app or smartphone apps or smartphone application or smartphone applications or mobile health apps or mobile health app or mobile health application or eHealth app or eHealth apps or eHealth applications or eHealth application or mobile apps or mobile application or mobile app or mobile applications or mobile phone app or mobile phone application or mobile phone apps or mobile phone applications or cell phones or cell phone or cell phone app or cell phone apps or cell phone application or cell phone applications)
Phenomenon of Interest	(smoking cessation or drinking reduction or harmful drinking or harmful drinkers or risk drinking or risk drinkers or problem drinkers or problem drinking or excess drinking or drinking in excess or excessive drinking or binge drinking or binge drinkers or drinking cessation or at risk drinkers or lifestyle or healthy eating or eating behaviours or diet or weight control or weight management or mindfulness or meditation or stress reduction or relax or relaxed or relaxing or relaxation or sleep hygiene or sleep habits or sleep behaviour or sleep quality or personal care or wellness or self-care or calorie intake or calorie reduction or weight change or fitness or exercise or exercises or exercising or healthy living or eating habits)
Evaluation	(obstacle or barrier or issues or obstruction or difficulties or difficulty or deter or deterrent or drawback or hinder or hinderance or hindering or prevents or preventing or prevention or impede or problem or complication or impediment or enables or enable or enabling or enabler or aid or aiding or promote or promoting or promotion or encourage or encouraging or encouragement or help or helping or assist or assisting or ease or easing or facilitators or facilitates or facilitating or empowers or empower or empowering or empowerment)
Design and Research Type	(qualitative or ethnograph or phenomenal or grounded theory or hermeneutic or observation or focus group or focus groups or interview or mixed methods or mixed method or multimethod or multi-method)

**Table A2:** CART Criteria.

**C: completeness**	Articles were eliminated if: - The intervention was not fully described. - The data were insufficient to address the review’s question.
**A: accuracy**	Articles were eliminated if: - They did not address a clear objective through qualitative measures. - If the data were insufficient to address the review’s question.
**R: relevance**	Articles were included only if: - Findings were relevant and deemed generalisable enough to extend to a healthy population of mHealth-app users. Due to the COVID-19 pandemic meaning that the digital health world is ever changing and evolving, it was deemed necessary to exclude articles where the findings were influenced significantly by the pandemic.
**T: timeliness**	Data had to be collected between 2016–2020.

## Appendix B. Critical Appraisal

**Table A3:** CASP Table.

	Wei Peng et al., 2016	Simons et al., 2018	Krishnan and Lokachari, 2019	Ming Li Carol Seah et al., 2021	Zhou et al., 2019	Kanthwala, 2019	Nurmi et al., 2020	Jones et al., 2019	Wu et al., 2017	Lyzwinski et al., 2018	Leffers. J, 2018	Darcel et al., 2018	Baretta, Perski & Steca, 2019	Nikolaou et al., 2019	Subasinghe et al., 2019	Xiaoxiao et al., 2020	Vaghefi and Tulu, 2019	Castro et al., 2020	Chandler et al., 2020	Chen, 2018	Nour et al., 2017	LaPrincess et al., 2019	Laurie, Blandford, 2016	Leffers J et al., 2018	Rose 2020
Was there a clear statement of the aims of the research?	C	Y	Y	Y	Y	Y	Y	Y	Y	C	Y	Y	Y	Y	Y	Y	Y	Y	Y	Y	Y	Y	Y	Y	Y
Is a qualitative methodology appropriate?	Y	Y	Y	Y	Y	Y	Y	Y	Y	C	Y	Y	Y	Y	Y	Y	Y	Y	Y	Y	Y	Y	Y	Y	Y
Was the research design appropriate to address the aims of the research?	Y	Y	Y	Y	Y	Y	Y	Y	C	Y	Y	Y	Y	N	Y	Y	C	Y	Y	Y	Y	Y	N	C	Y
Was the recruitment strategy appropriate to the aims of the research?	N	C	Y	Y	Y	Y	Y	Y	Y	Y	Y	Y	Y	Y	Y	Y	Y	Y	N	Y	Y	Y	Y	Y	C
Was the data collected in a way that addressed the research issue?	Y	Y	Y	C	Y	C	Y	Y	Y	Y	N	Y	Y	Y	Y	Y	N	Y	Y	Y	Y	N	Y	Y	Y
Has the relationship between researcher and participants been adequately considered?	N	C	N	Y	Y	Y	N	Y	Y	Y	Y	N	C	Y	C	N	N	C	Y	N	Y	N	Y	Y	N
Have ethical issues been taken into consideration?	Y	Y	C	Y	Y	C	Y	Y	Y	C	Y	Y	Y	Y	Y	Y	Y	Y	Y	C	Y	C	Y	Y	Y
Was the data analysis sufficiently rigorous?	Y	Y	Y	Y	Y	C	Y	C	C	Y	Y	Y	Y	Y	Y	Y	Y	Y	Y	Y	Y	Y	Y	Y	Y
Is there a clear statement of findings?	Y	Y	Y	Y	Y	Y	Y	Y	Y	Y	Y	Y	Y	Y	Y	Y	Y	Y	Y	Y	Y	Y	Y	Y	Y
Is the research valuable	Y	Y	N	Y	Y	Y	Y	Y	Y	Y	Y	Y	Y	Y	Y	Y	Y	Y	Y	Y	Y	Y	Y	Y	Y

Table for the CASP (Critical Appraisal Skills Programme) Qualitative Checklist: Y = Yes, C = Can’t tell, N = No.

**Table A4:** Critical Appraisal Table.

Author & Year	Method	Sample Characteristics	Critical Appraisal
Nikolaou et al., 2019	Survey + focus groups	Survey, *n* = 2285 Focus group, *n* = 719 young individuals aged 13–24	Unrepresentative sample: Global study in 6 countries but only conducted focus groups in English. Therefore, only those capable of speaking English recruited, which may reflect high educational standards; thus, views may not necessarily be reflective of non-English speakers in those countries.
Chandler R. et. al, 2020	Focus groups	*n* = 23	Small sample size.
			Poor recruitment methods -> Only one of the 4 focus groups targeted the ideal age group for the proposed mobile app.
			Black women involved all from a similar community; therefore, results of this study can only be generalized to women of a similar demographic.
			Only focus groups used; therefore, high susceptibility to biases such as halo effect and the dominance effect.
Laurie and Blandford, 2016	Semi-structured interviews	*n* = 16	Study design limits the data that could have been collected: i.e., data were only gathered at start and end of study, meaning that experiences during the actual study were not looked into.
			HAWTHORNE EFFECT Lack of consistency in exit interview technique, i.e., later changed to become more open and participant directed.
			Small sample size. Unrepresentative: results cannot be generalised in context of wider population. All participants lived in London and had similar employment commitments; 11 out of 16 participants were female.
Nour.M et al., 2017	Focus group	Focus group *n* = 32	Solely focus-group-based methodology; therefore, vulnerable to biases such as groupthink bias.
			Not generalizable to other age groups or individuals from different countries to Australia where culture may be different.
Nurmi et al., 2020	Think aloud and semi-structured interviews	*n* = 12	Small sample size, not representative of wider population.
			Interviews only provided information on participants’ initial reactions to the app’s features.
			Inconsistency in what was presented to individuals (app was being developed so later participants had more features to form views from).
			Researcher-bias: Researchers prompted participants when they were silent for a prolonged period of time may introduce bias.
Régnier, Dugré, Darcel et al., 2018	Questionnaires + 2 sets of Semi-structured Interviews	(pre-app use) *n* = 46 questionnaires to help design app *n* = 12 in depth interviews (users of app) *n* = 21 supplementary interviews (low-income individuals in general population)	Small sample size.
			Choice of the participants was limited to individuals from underprivileged backgrounds. These individuals had a very heavy combination of constraints (e.g., budget, social integration, and language).
Lieffers. J et al., 2018	Semi-structured interviews	*n* = 24	Small sample size.
			Unrepresentative of population: skewed sample with many participants being female and 18–30 years of age and participants being recruited from two Canadian provinces.
			Lack of demographic information of participants collected; therefore, difficult to determine generalizability of results.
Wei Peng et al., 2016	Interviews, focus groups	*n* = 44	Only 5 participants outside of the university setting (lack of diversity amongst participants). These 5 participants were interviewed individually as opposed to in a focus group due to time constraints (inconsistent methodology).
			Focus groups may introduce several biases including the halo effect.
			2/3 of the participants were female.
			Not all the participants had used health apps before, and thus the discussion was based on their perceptions from examples provided by the trigger materials, which may be inconsistent from their perceptions if they actually had used health apps.
Wu et al., 2017	Semi-structured interviews (think aloud)	*n* = 10	No quantitative data to support engagement measurements. Not generalisable to other digital interventions. Small sample size. Input (despite being think aloud) from the interviewer might have influenced the users. Participants were compensated.
Vaghefi and Tulu, 2019	Interviews and diary entries	Pre-use and post-use interview: 17 participants, 193 diaries from same 17 participants	More than half of the participants were female, used an iPhone, and were highly motivated to take care of their health—generalisability might be called into question, especially if you consider income and pre-usage motivation as factors. Extremely subjective in terms of data collected—would have been better if there was objective data to compare against. Only focused on health and wellness apps—not extended to other types of apps. Participants were compensated and all came from the same environment (university). Sent daily reminders to users to remind them to log in the diary—their influence was not kept to a minimum. Small sample size.
Jones et al., 2019	Focus groups	Focus group, *n* = 15	Small sample size.
			Only mothers included-not fathers/other carers, e.g., grandparents. From 1 geographical area.
Castro et al., 2020	Focus groups	*n* = 17	Enrolled only people over 40 with high social vulnerability—did not compare across different demographics and incomes. Active people spoke more in the focus group than the non-active people; therefore, the design of the app will be skewed towards preferences of active rather than non-active participants.
LaPrincess C. Brewer, 2019	Focus groups and survey	Focus group 23, co-design 13, survey 36	Small sample size.
			Most participants are women (but this is inherent to AA church).
Ming Li Carol Seah et al., 2021	Focus-group interviews	*n* = 8	App was only used for 3 days.
			Small sample size of university students’ perspectives on mindfulness and mHealth.
Subasinghe et al., 2019	Focus-group questionnaire	Focus group = 4 questionnaire = 23	Greater education and older participants are over-represented. Small sample size. Short follow up in usability.
Lieffers, J., 2018	Interviews	*n* = 26	Small sample size.
			Way interviewees were recruited (email to users) means that they are more likely to give feedback.
			Primarily female and 18–50 years of age; however, this distribution generally reflects the overall population of eaTracker users.
			Data on education level, income, and ethnicity were not collected from participants.
Huan Chen, 2018	Interview	*n* = 20	The data are specific to the culture (Chinese) and may not be generalizable. All citizens came from urbanised locations, so the barriers and facilitators might be different.
Xiaoxiao et al., 2020	Questionnaire, interviews	Questionnaire *n* = 379 Interviews *n* = 10	Sample is unrepresentative (mostly female, college-educated, and tech-savvy). The cross-sectional data do not allow for the analysis of predictive power in understanding people’s post-adoptive behaviours
Dario Baretta, Olga Perski, Patrizia Steca, 2019	Semi structured interviewed	Baseline n = 20, after 2 weeks *n* = 17	Heterogeneity amongst digital-literacy abilities of the sample group.
Kanthawala, S.	Semi-structured interview (think-aloud)	Interviews: *n* = 19	Participants were highly educated and from the same city. Digital literacy was not assessed.
Krishnan and Lokachari, 2019	Focus groups, interviews	Focus Groups: *n* = 12, Interview: *n* = 12	Dominance effect (a dominant individual shapes the discussion), halo effect (the perceived status of a group member influences the discussion), and groupthink (the members in a group tend to think similarly to maintain group cohesion), among several others (Mukherjee et al., 2015). Not generalisable to other age groups. Needs to be backed up by empirical data.
Zhou et al., 2019	Questionnaire, semi-structured interview	*n* = 117 (survey), *n* = 117 (semi-structured interviews)	Not generalisable to the entire population (e.g., sick and the elderly). Not applicable to other countries where regulations/cultures might be different.
Simons et al., 2018	Focus groups, semi-structured interviews	Interviews *n* = 10 Focus groups *n* = 34	App is specifically adapted to the Flemish lower-educated working young adults, which limits its generalizability.
Lynnette Nathalie Lyzwinski et al., 2019	Focus group, written feedback	*n* = 8	This is a single qualitative exploratory study with a participatory pilot design at one university campus with a mostly female sample.
			The purposive nature of the study required some previous experience with general health apps and some idea of mindfulness, which limited the pool of interest and eligibility. Funding limitations also restricted this to a small pilot participatory study.
Johanna Friederike Rose 2020 May 2020	Interviews	*n* = 11	Small sample size: All highly educated, higher proportion of men, recruited via snowball technique, all between 22–28.
			Furthermore, studying people that a researcher knows personally might lead to compromises in the researcher’s ability to disclose information and raises issues of an imbalance of power between the inquirer and the participants. Interviewees first language varied—communication barriers

## References

[1] Mobile for Development The State of Mobile Internet Connectivity Report 2020. https://www.gsma.com/r/somic/.

[2] Vaghefi I., Tulu B. (2019). The Continued Use of Mobile Health Apps: Insights from a Longitudinal Study. JMIR mHealth uHealth.

[3] Nouri R., Kalhori S.R.N., Saeedi M.G., Marchand G., Yasini M. (2018). Criteria for assessing the quality of mHealth apps: A systematic review. J. Am. Med. Inform. Assoc..

[4] Pires I.M., Marques G., Garcia N.M., Flórez-Revuelta F., Ponciano V., Oniani S. (2020). A Research on the Classification and Applicability of the Mobile Health Applications. J. Pers. Med..

[5] Castle-Clarke S., Imison C. (2016). The Digital Patient: Transforming Primary Care.

[6] McKay F.H., Wright A., Shill J., Stephens H., Uccellini M. (2019). Using Health and Well-Being Apps for Behavior Change: A Systematic Search and Rating of Apps. JMIR Mhealth Uhealth.

[7] Cooke A., Smith D., Booth A. (2012). Beyond PICO: The SPIDER tool for qualitative evidence synthesis. Qual. Health Res..

[8] Moher D., Liberati A., Tetzlaff J., Altman D.G. (2009). Preferred reporting items for systematic reviews and meta-analyses: The PRISMA statement. BMJ.

[9] Jahns R.-G. The mHealth Apps Market Is Getting Crowded. 2017. pp. 1–6. https://research2guidance.com/mhealth-app-market-getting-crowded-259000-mhealth-apps-now/.

[10] Baretta D., Perski O., Steca P. (2019). Exploring Users’ Experiences of the Uptake and Adoption of Physical Activity Apps: Longitudinal Qualitative Study. JMIR mHealth uHealth.

[11] Guest G., MacQueen K., Namey E. (2012). Applied Thematic Analysis.

[12] Kumar A., Mhaskar R., Emmanuel P., Mishra S., Patel S., Naik E. (2009). Critical appraisal skills are essential to informed decision-making. Indian J. Sex. Transm. Dis. AIDS.

[13] Zhang X., Xu X. (2020). Continuous use of fitness apps and shaping factors among college students: A mixed-method investigation. Int. J. Nurs. Sci..

[14] Laurie J., Blandford A. (2016). Making time for mindfulness. Int. J. Med. Inform..

[15] Peng W., Kanthawala S., Yuan S., Hussain S.A. (2016). A qualitative study of user perceptions of mobile health apps. BMC Public Health.

[16] Castro P.C., Romano L.B., Frohlich D., Lorenzi L.J., Campos L.B., Paixão A., Bet P., Deutekom M., Krose B., Dourado V.Z. (2020). Tailoring digital apps to support active ageing in a low income community. PLoS ONE.

[17] Lyzwinski L.N., Caffery L., Bambling M., Edirippulige S. (2018). University Students’ Perspectives on Mindfulness and mHealth: A Qualitative Exploratory Study. Am. J. Health Educ..

[18] Seah M.L.C., Koh K.T. (2020). The efficacy of using mobile applications in changing adolescent girls’ physical activity behaviour during weekends. Eur. Phys. Educ. Rev..

[19] Simons D., De Bourdeaudhuij I., Clarys P., De Cocker K., Vandelanotte C., Deforche B. (2018). A Smartphone App to Promote an Active Lifestyle in Lower-Educated Working Young Adults: Development, Usability, Acceptability, and Feasibility Study. JMIR mHealth uHealth.

[20] Wu J., Tombor I., Shahab L., West R. (2017). Usability testing of a smoking cessation smartphone application (‘SmokeFree Baby’): A think-aloud study with pregnant smokers. Digit. Health.

[21] Nour M., Chen J., Allman-Farinelli M., Leung M., Fazzino T. (2019). Young Adults’ Engagement with a Self-Monitoring App for Vegetable Intake and the Impact of Social Media and Gamification: Feasibility Study. JMIR Form. Res..

[22] Gerke S., Stern A.D., Minssen T. (2020). Germany’s digital health reforms in the COVID-19 era: Lessons and opportunities for other countries. NPJ Digit. Med..

[23] Régnier F., Dugré M., Darcel N., Adamiec C. (2018). Providing a Smart Healthy Diet for the Low-Income Population: Qualitative Study on the Usage and Perception of a Designed Cooking App. JMIR mHealth uHealth.

[24] Krishnan G., Lokachari P.S. Adoption of health and fitness apps by mobile users: Interactive qualitative analysis. Proceedings of the 2019 Portland International Conference on Management of Engineering and Technology (PICMET).

[25] Nikolaou C.K., Tay Z., Leu J., Rebello S.A., Morenga L.T., Van Dam R.M., Lean M.E.J. (2019). Young People’s Attitudes and Motivations Toward Social Media and Mobile Apps for Weight Control: Mixed Methods Study. JMIR mHealth uHealth.

[26] Rose J. (2021). Using the Bad for Something Good” Exploring the Possible Paradox of Meditation Apps in Light of Digital Stress. DIVA. http://www.diva-portal.org/smash/record.jsf?pid=diva2%3A1446414&dswid=6017.

[27] Brewer L.C., Hayes S.N., Caron A.R., Derby D.A., Breutzman N.S., Wicks A., Raman J., Smith C.M., Schaepe K.S., Sheets R.E. (2019). Promoting cardiovascular health and wellness among African-Americans: Community participatory approach to design an innovative mobile-health intervention. PLoS ONE.

[28] Lieffers J.R., Arocha J.F., Grindrod K., Hanning R.M. (2017). Experiences and Perceptions of Adults Accessing Publicly Available Nutrition Behavior-Change Mobile Apps for Weight Management. J. Acad. Nutr. Diet..

[29] Kanthawala S., Joo E., Kononova A., Peng W., Cotten S. (2018). Folk theorizing the quality and credibility of health apps. Mob. Media Commun..

[30] Jones F., Whitehouse A., Dopson A., Palaghias N., Aldiss S., Gibson F., Shawe J. (2019). Reducing unintentional injuries in under fives: Development and testing of a mobile phone app. Child Care Health Dev..

[31] Nurmi J., Knittle K., Ginchev T., Khattak F., Helf C., Zwickl P., Castellano-Tejedor C., Lusilla-Palacios P., Costa-Requena J., Ravaja N. (2020). Engaging Users in the Behavior Change Process With Digitalized Motivational Interviewing and Gamification: Development and Feasibility Testing of the Precious App. JMIR mHealth uHealth.

[32] Chandler J., Cumpston M., Thomas J. (2021). Cochrane Handbook for Systematic Reviews of Interventions. https://training.cochrane.org/handbook.

[33] Subasinghe A.K., Garland S.M., Gorelik A., Tay I., Wark J.D. (2019). Using Mobile Technology to Improve Bone-Related Lifestyle Risk Factors in Young Women with Low Bone Mineral Density: Feasibility Randomized Controlled Trial. JMIR Form. Res..

[34] Zhou L., Bao J., Watzlaf V., Parmanto B. (2019). Barriers to and Facilitators of the Use of Mobile Health Apps from a Security Perspective: Mixed-Methods Study. JMIR mHealth uHealth.

[35] Pampel F.C., Krueger P.M., Denney J.T. (2010). Socioeconomic Disparities in Health Behaviors. Annu. Rev. Sociol..

[36] Loring B., Robertson A. (2021). Obesity and Inequities. https://www.euro.who.int/__data/assets/pdf_file/0003/247638/obesity-090514.pdf.

[37] Lucivero F., Jongsma K.R. (2018). A mobile revolution for healthcare? Setting the agenda for bioethics. J. Med. Ethics.

[38] Hurst L., Mahtani K., Pluddemann A., Lewis S., Harvey K., Briggs A., Boyle A., Bajwa R., Haire K., Entwistle A. (2019). Defining Healthcare in the NHS: CEBM Report. https://www.cebm.net/2019/04/defining-value-based-healthcare-in-the-nhs/%0A1.

[39] Azuma K., Nojiri T., Kawashima M., Hanai A., Ayaki M., Tsubota K., TRF-Japan Study Group (2021). Possible favorable lifestyle changes owing to the coronavirus disease 2019 (COVID-19) pandemic among middle-aged Japanese women: An ancillary survey of the TRF-Japan study using the original “Taberhythm” smartphone app. PLoS ONE.

[40] Di Domenico S.I., Ryan R.M. (2017). The Emerging Neuroscience of Intrinsic Motivation: A New Frontier in Self-Determination Research. Front. Hum. Neurosci..

[41] Rohan A. (2021). Has Lockdown Made Consumers More Open to Privacy. Ernst & Young. https://www.ey.com/en_uk/consulting/ey-global-consumer-privacy-survey/has-lockdown-made-consumers-more-open-to-privacy.

[42] Sydow L. (2021). The Impact of Coronavirus on the Mobile Economy as Businesses Enforce Working from Home, Conferences are Postponed, Flights are Canceled, Schools are Closed, Storefronts and Restaurants Shut Their Doors and People are quarantined Inside, the World Is. https://www.appannie.com/en/insights/market-data/coronavirus-impact-mobile-economy/.

[43] NHS England (2020). Around One Million Downloads of Fitness App during Lockdown as People Stay Fit. https://www.england.nhs.uk/2020/07/around-one-million-downloads-of-fitness-app-during-lockdown-as-people-stay-fit/.

